# It Cost Us All of Our Savings to Deliver Our Baby: A Qualitative Study to Explore Barriers and Facilitators of Maternal and Child Health Service Access and Utilization in a Remote Rural Region in India During the COVID-19 Pandemic

**DOI:** 10.7759/cureus.35192

**Published:** 2023-02-19

**Authors:** Subhanwita Manna, Saurav Basu

**Affiliations:** 1 Indian Institute of Public Health-Delhi, Public Health Foundation of India, New Delhi, IND

**Keywords:** covid-19 in pregnancy, coronavirus pandemic, sars-cov-2, maternal-child health, covid-19

## Abstract

Introduction: During the coronavirus disease 2019 (COVID-19) pandemic, rural and geographically isolated regions in lower-middle-income countries (LMICs) encountered major deficits in maternal and child health (MCH) care that were accentuated by pre-existing weak public health infrastructure and diversion of existing health resources for pandemic management purposes. This explorative qualitative study was conducted to assess the barriers, challenges, and facilitators in the access and utilization of essential MCH services among pregnant women during the COVID-19 pandemic in a geographically remote and rural area in India, having nearly 70% rural population.

Method: The study was conducted using an ethnographic approach. Three villages were selected purposively from the Purba Medinipur district of the Eastern state of West Bengal, geographically isolated by a local river. Information on challenges of utilization was collected by in-depth interviews (IDI) with a universal sample of 25 mothers who underwent pregnancy after March 2020 and focus group discussions (FGD) with their husbands and mothers-in-laws. Thematic analysis was used to analyze the qualitative data.

Results: The median (IQR) age of the mothers that delivered during pregnancy were 23 (18, 28) years and ranging from 18 to 28 years (N=25). All the mothers were married, housewives, literate, and Hindu by religion, while in the accompanying husband cohort, a majority (56 %) had completed high school. Half (52%) were primigravida with at least one living child (60 %). All the mothers had a successful birth outcome and only one had current evidence of mild depression. Low utilization of MCH services during the pandemic in the study area was recognized as an outcome of individual-level, interpersonal-level, and community-level barriers. Diversion of routine health staff for COVID-19 related services occasionally compelled pregnant women and children to seek care from unlicensed healthcare providers who remained accessible even during periods of stringent lockdown. Furthermore, the irregular functioning of the local primary health care system translated into missed home visits and disruption of nutritional assistance services. A dual burden of economic loss was reported in most households from loss of livelihood and wages and additional expenditure incurred in underdoing deliveries at private health facilities, thereby potentially translating into catastrophic out-of-pocket costs. The designation of a separate government health facility for delivery due to the unavailability of the local hospital did not mitigate the circumstances due to its lack of utilization by the villagers who encountered difficult access and a lack of trust in an unfamiliar environment. The functioning of a popular conditional cash transfer scheme for promoting safe motherhood was also possibly compromised during the pandemic.

Conclusions: Accessibility to MCH services was severely affected during the COVID-19 pandemic, especially during the stringent lockdown periods in remote and rural areas in India. Future pandemic preparedness must have enhanced health policy and administrative focus on preventing significant disruption of MCH services by maintaining improved accessibility to alternative health facilities, monitoring regular home visits by frontline health workers, rendering effective distribution of benefits from existing social protection schemes, and universal promotion of respectful maternity care.

## Introduction

Maternal and child morbidity and mortality due to deficient maternal and child health (MCH) services is a major public challenge in lower-middle-income countries (LMICs) including India, a country with nearly 70% rural population. During the COVID-19 pandemic, accessibility, availability, and utilization of MCH services were significantly compromised due to the interruption of the continuum of care necessary for maintaining antenatal care, safe institutional delivery, and postpartum care services. Rural and geographically isolated remote areas in LMICs experienced even greater vulnerability due to their pre-existing limited primary care infrastructure, and the diversion of the existing health staff for pandemic management tasks [1]. Furthermore, lack of transportation, stigma, and fear of infection during periods of stringent lockdown further restricted the accessibility of healthcare services. It is estimated that the Asia-Pacific region experienced a 20-50% drop in the availability of essential maternal healthcare services, especially antenatal care, and institutional delivery during the pandemic which was primarily driven by shortages of infrastructure and healthcare workers [2]. In India, the female frontline health workers known as the Accredited Social Health Activists (ASHAs) are also the local link workers connecting the rural primary health system with their communities [3]. The Village Health Sanitation and Nutrition Committee (VHSNC) is another village-level structure, that was established in 2007 to monitor the monthly Village Health Sanitation and Nutrition Day (VHSND) and health services, particularly MCH and nutrition services [1,4].

In addition, auxiliary nurse midwives (ANMs) are the grass-root level workers that provide services at first referral units and perform routine deliveries at primary care facilities. However, during the pandemic period, frontline and community health workers were channelized in pandemic-related assignments like surveillance, contact tracing, and COVID-19 vaccination which is likely to have affected the routine MCH service delivery [5,6]. India has achieved success in reducing the maternal mortality rate to 113 in 2018 from 166 in 2013 [7] through the strengthening of MCH services although it is still significantly lagging behind the sustainable development goal targets. Furthermore, there exists considerable state-wise heterogeneity in the quality of primary care services in India [8]. We conducted this explorative qualitative study to assess the barriers, challenges, and facilitators in the access and utilization of essential MCH services in pregnant women during the COVID-19 pandemic in a geographically remote and rural area in India.

## Materials and methods

Study design and setting

This study was conducted in the Eastern state of West Bengal in India which has moderate-low health-related parameters as per the District Level Household and Facility Survey (DLHS) index findings apart from high out-of-pocket expenditures [9,10].

**Figure 1:**
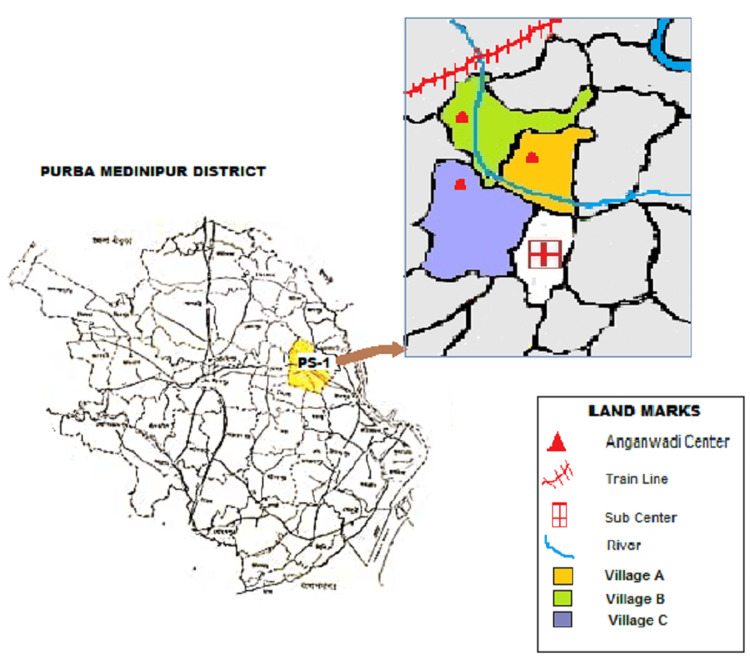
Geographical location of study setting

An ethnography approach was used to collect data on MCH service utilization and its challenges amongst women who experienced pregnancy since the pandemic period in three villages named Tikarpara (Village-A), Gobindacak (Village-B), Atang (Village-C) of Purba Medinipur District, West Bengal. These villages have a total population of 530, 492, and 687 people, respectively. One sub-center was selected purposively considering its proximity to the hospital. Three of the most geographically inaccessible villages were subsequently selected from amongst the 10 villages catered by the Kumarpur sub-center due to their geographic isolation by a local river Khiri (Figure 1).

According to Health Management Information System (HMIS), in the present study block (Panskura-I), 63.78% (2327/3649) of pregnant women, who had registered their pregnancy in any government facilities in the area also availed institutional delivery at similar institutions during the pre-pandemic period (2018-2019). However, during the pandemic period (2019-2020) the rate of delivery at government facilities in the area was reduced to 62.18% (2116/3403). The Uttar Mechogram Super Speciality Hospital, a former block primary health center, is the solitary government hospital offering delivery services to the residents of the village in the study area. During the COVID-19 pandemic, this facility was predominantly used for the management of COVID-19 patients which precluded its use for other conditions including both normal deliveries and caesarian sections. The only health camp that serves the two geographically distant villages, known as Village Health, Sanitation and Nutrition Day (VHSND) camp, is hosted at Anganwadi center on a fixed day of every month to guarantee easy accessibility and availability of MCH services was moved to a different site. A different primary health center (PHC), located about 15 km from the super specialty hospital was designated as the preferred site for continuing MCH services for the women from the study area. 

Sample and sampling technique

A total of 25 women were selected using the universal sampling approach. Women who were permanent residents of the selected villages and gave birth to a live baby between April 1, 2020, and December 31, 2021, were chosen to understand MCH service utilization and the issues they and their families faced during and following the lockdown. Mothers-in-law and spouses are also enlisted as study participants in order to gain a better understanding of the MCH services offered at the village level, as well as the factors that influence service utilization and accessibility. The researchers conducted the interviews during home visits. The mother-in-law and spouse were also selected during the home visit and were requested to report for the focus-group discussions (FGDs) to the local village clubhouse in Village-A on fixed dates.

Data collection procedure

The primary approaches for gathering data were in-depth interviews (IDIs) and FGDs, both of which were followed by field notes. There were 25 IDIs conducted with pregnant and lactating mothers. Each interview lasted between 30-60 mins, ending when data saturation was attained. Each focus group discussion lasted 60-90 mins, and it was assumed that the conversation had concluded when no new topics or ideas seemed to emerge. The FGD participants were husbands and mothers-in-law from the three study areas. To prevent the potential transmission of COVID-19 among FGD discussants, a number of safety measures were put in place. These included maintaining physical distance, donning masks, and restricting the number of FGD groups to no more than eight. The focus groups were moderated by the researcher herself.

Research instruments

A structured interview schedule was used to gather information on the sociodemographic characteristics of women, and the government-approved Mother and Child Protection (MCP) card was used to validate the data. However, the women's height, weight, and mid upper arm circumference (MUAC) were assessed using standardized techniques and tools, and postpartum depression was assessed using the Edinburgh Postnatal Depression Scale (EPDS) [11,12]. IDI and FGDs were conducted to gather qualitative data.

The study tools were initially created in English in collaboration with a group of public health experts to ensure face validity. Two people who were fluent in both languages used the suggested forward translation approach to linguistically validate the English tool in Bengali, the native language.

Data analysis

**Figure 2:**
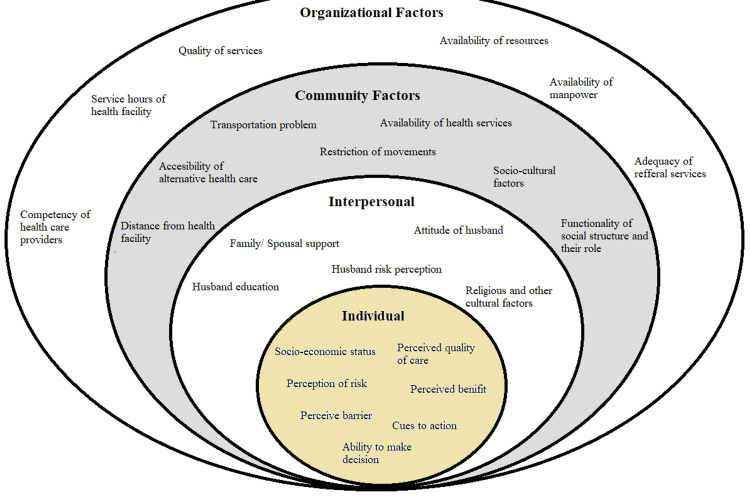
Socio-Ecological model of the utilization of MCH services MCH: Maternal and child health

The information was gathered in its native language, Bengali, and afterward translated into English for analysis. The influence of COVID-19 on the use of MCH services in rural West Bengal was conceptualized using the social-ecological model (SEM) (Figure 2). The SEM offers a theory-based method for comprehending the intricate interactions between institutional, policy, interpersonal, community, and individual factors that affect behavior and practice [13].

Thematic analysis was performed on the data collected from FGDs and IDIs using QDA Miner Lite 4.0 software [14]. The transcribed data was matched to the audio recordings and notes to ensure accuracy. The transcripts were read, deciphered, and coded. A-priori and data-driven themes were combined with similar codes to create umbrella themes. Two investigators reviewed the transcripts to methodically classify the coded data under the major discussion points, allowing main themes to emerge from each topic. The final themes were named and defined after the thematic consensus was double-checked for any inconsistencies.

Ethical approval

The study was approved by the Institutional Ethics Committee (IEC) of the Indian Institute of Public Health, Delhi (Reference no IIPH_IEC_S-35_2021). Administrative permission was obtained from the Panchayet Pradhan of Haur Gram Panchayet (Memo No- 247/HGP). All participants provided written and informed consent to participate in this study.

## Results

Socio-demographic profile of the participants 

A total of 25 mothers were included in this study and their sociodemographic characteristics are reported in Table 1. The median (interquartile range {IQR}) age of the mothers was 23 (18, 28) years. All the mothers were married, housewives, literate, and Hindu by religion.

**Table 1:** Socio-demographic characteristics of participants ST: Scheduled tribes; OBC: Other backward classes

Characteristics	Frequency (n)	Percentage (%)
Age (years)		
18-23	14	56
24-30	11	44
Religion		
Hindu	25	100
Caste		
General	19	76
ST	2	8
OBC	4	16
Educational status of women		
No education	0	0
Up-to Class V	4	16
Class VI - XII	15	60
Graduation	3	12
Post graduation and above	3	12
Educational status of Husband		
No education	1	4
Up-to Class V	7	28
Class VI - XII	14	56
Graduation	2	8
Post graduation and above	1	4
Occupation		
Housewife	25	100
Occupation of husband		
Agricultural workers	6	24
Daily wage workers	4	16
Self employed	10	40
Govt salaried	4	4
Private salaried	4	16
Family type		
Joint	3	12
Nuclear	3	12
Extended	19	76
Family income (per month)		
Less than 15000	13	52
Above 15000	12	48
Mass media exposure		
Yes	16	64
No	9	36

Among the cohort of husbands, a majority (56 %) had completed high school. The majority of the women (52%) were primigravida with at least one living child (60%). All the participants had a successful birth outcome, while only 8% of them developed complications during pregnancy for which they did not consult with any healthcare worker (Table 2). Only one participant had evidence of mild depression measured with the Edinburgh Postnatal Depression Scale (EPDS).

**Table 2:** Obstetric and health characteristics of the participants ^a ^EPDS: Edinburgh Postnatal Depression Scale

Characteristics	Total (N=25) n,(%)
Pregnancy type	
Primipara	13 (52)
Multipara	12 (48)
History of abortion	
0	23 (92)
1	2 (8)
Number of living children	
1	15 (60)
≥2	10 (40)
Age of the last child (months)	
0-12	14 (56)
12-24	11 (44)
Pregnancy compilation	
Yes	2 (8)
No	23 (92)
Consultation of complications with healthcare workers	
Yes	0 (0)
No	25 (100)
Body Mass Index (Median, IQR)	22.5 (19.1, 25.9)
EPDS score^a^	
No depression (0-6)	24 (96)
Mild depression (7-13)	1 (4)
Mid Upper Arm Circumference (MUAC)	
Severe undernourished (<19)	0 (0)
Mild to moderate undernourished (19-22)	3 (12)
Normal (23 and above)	22 (88)

All the women registered their pregnancy in a government facility. Among the women who were pregnant or delivered during the period of lockdown (March-May 2020 and May 2021), only one (14.3 %) (n=7) received the minimum recommended four antenatal care (ANC) checkups whereas those who were pregnant or delivered after lockdown, all (100 %) received at least four ANC checkups (n=18). During the study period, 22 participants gave birth in a private facility, two gave birth in a government facility outside the area, and one participant had a home delivery (Table 3).

**Table 3:** Utilization of MCH services MH: Maternal and child health

Characteristics	Pregnant and delivered after lockdown (N=18) n %	Pregnant and delivered during lockdown (N=7) n %	Total (N=25) n %
Pregnancy registration			
Yes	18 (100)	7 (100)	25 (100)
Number of Antenatal Care checkups			
1	0 (0)	1 (14.3)	1 (4)
2	0 (0)	3 (42.9)	3 (12)
3	0 (0)	2 (28.6)	2 (8)
4	18 (100)	1 (14.3)	19 (76)
>=100 Iron Folic Acid Supplementation			
Yes	17 (94.4)	7 (100)	24 (96)
No	1 (5.6)	0 (0)	1 (4)
Tetanus toxoid injection			
2 doses	18 (100)	7 (100)	25 (100)
Contact with healthcare workers			
Over phone	0 (0)	6 (85.7)	18 (82)
During home visits	18 (100)	1 (14.3)	7 (28)
Received pregnancy and delivery related information			
Yes	16 (88.9)	2 (28.6)	18 (82)
No	2 (11.1)	5 (71.4)	7 (28)
Place of delivery			
Home	0 (0)	1 (14.3)	1 (4)
Govt hospital	2 (11.1)	0 (0)	2 (8)
Private hospital	16 (88.9)	6 (85.7)	22 (88)
Type of delivery			
Caesarean section	16 (88.9)	6 (85.7)	22 (88)
Normal Vaginal Delivery	2 (11.1)	1 (14.3)	3 (12)
Delivery assistance			
Trained personnel	18 (100)	6 (85.7)	24 (96)
Local quack	0 (0)	1 (14.3)	1 (4)
No of Postnatal Care visits			
0-2	0 (0)	3 (42.9)	3 (42)
3-5	18 (100)	4 (57.1)	22 (88)
Child immunization according to age			
Yes	16 (88.9)	3 (42.9)	19 (76)
No	2 (11.1)	4 (57.1)	6 (24)

Following the coding of the interview data, four key themes became apparent as having a substantial influence on the use and accessibility of MCH services which are described below.

A. Utilization of services during the COVID-19 pandemic: continuity of care during and after lockdown

There was a significant service utilization gap in accessing ANC, prenatal care (PNC), and sick newborn care services since most of the existing healthcare providers were diverted for pandemic management activities. Restricted mobility with hard transport access especially during periods of severe lockdown and limited primary health system functioning major contributed to the difficulty in healthcare accessibility. Anganwadi services were halted, and village health, sanitation, and nutrition day (VHSND) camps were sporadically conducted throughout the lockdown period which caused difficulty for pregnant and lactating women (PLW) and infants in receiving nutritional assistance. Post-lockdown, the government hospital continued to function as a COVID-19 management facility with a continued suspension of delivery services. 

B. Barrier in MCH service utilization during the pandemic

Theme-1: Individual Level Barriers

The double burden of the financial crisis (Economic hardship and MCH related out of pocket expenses): During the pandemic, most villagers employed in the informal sector experienced significant economic losses and unemployment. Most participants reported incurring high out-of-pocket expenses for meeting their MCH needs during the pandemic due to difficult government health facility access and lack of pre-existing health insurance to purchase medications, investigations, and inpatient admissions.

“I was pregnant during the pandemic. Because the government hospital was a COVID hospital and all the subsidized services were unavailable, we were forced to pay every last rupee for care in a private facility. My caesarean delivery cost between 50,000-60,000/- nearly all of our savings”. (P6, Village-A, age 28 yrs, second gravida).

Fear of infection and unfamiliar settings: Participants were often unfamiliar with the location of the newly designated government health center for deliveries which combined with the long distance from their homes dissuaded its utilization.

“The government hospital is still functioning as a COVID hospital, so we were asked to visit another primary health care (PHC) centre in Purba Itara, almost 2-2.30 hours to reach. Instead, we visited a nearby private hospital for the delivery of my baby.” ( P1, husband, age 26 yrs).

Furthermore, some participants reported avoiding antenatal care appointments for fear of contracting COVID-19 infection. According to the only participant who had a home delivery:

“Second wave lockdown began while I was nine months pregnant. The situation was dreadful. The hospitals were overburdened with COVID-19-infected people, so I didn't want to risk putting my child in danger by going to a health facility. So, with the help of a local quack, I delivered my baby at home.” (P19, Village-B, age 25 yrs, second gravida).

"Our entire community was concerned about the virus. Because one of our neighbors was afflicted with the COVID-19 virus, my mother-in-law told me not to leave the house.” (P12, Village-B, 21 yrs, primigravida).

Reliance on unlicensed practitioners: Unlicensed rural medical practitioners were increasingly consulted by the participant's households for MCH-related problems during the pandemic lockdown due to the reduced frequency of household visits by frontline and community health workers. The unavailability of any pharmacy store in the village further accentuated the challenge of drug access. For example, one of the husbands reported his experience: 

“When my son had loose motions, I would request ASHA to give him medication …but she never did. As a result, I stopped consulting them concerning my wife's and child's health. My son was cured after I sought advice from our local quack.” (P8, husband, age 29 yrs).

Increase household responsibility: Many participants reported that restriction of movements during the pandemic encumbered young women to increase domestic chores which negatively influenced their health-seeking behavior.

“My husband and father-in-law, as well as my six-year-old child, were all at home during the lockdown. I had to complete all of the household chores while still meeting their demands. I didn't have time to go to the doctor for my regular antenatal checkups, so I skipped them” (P13, Village-B, 28 yrs, second gravida).

Theme-2: Interpersonal Level Barriers

Household decision making: Previous delivery experiences of other female family members were often an influential determinant of care-seeking decisions for current pregnancies in the household. The pregnant women reported that they along with their husbands and senior household members participated in an informed decision-making process.

“Due to the pandemic, the only government hospital was closed for non-COVID patients, so my mother-in-law advised me to deliver in the same hospital where my sister-in-law delivered her baby as the nursing home is good, clean and services are satisfactory.” (P23, Village-C, 20 yrs, primigravida mother) 

Theme-3: Community Level Barriers

Transportation and access barriers: Mothers complained about long distances and lack of reliable and affordable transportation facilities as reasons for their inability to visit the designated government health facility for accessing MCH services during the pandemic. For instance; 

“To avail the services of the sub-center either we have to cross a river, the connecting bridge is very risky to cross during this time or we have to cross the rail lines, which is equally risky, so I have to take her to the private practitioner for all the testing and checkups.” (P- 3, husband, age-30 yrs). Another participant reported: “There was no ambulance service available at that (pandemic/lockdown) time, so my husband booked a private cab to take me to the hospital. We were charged more than normal, but we didn't have any choice.” (P11, Village-B, age 19 yrs, second gravida)

Theme-4: Organizational Level Barriers

Preferred location of delivery in terms of perceived quality of care, risk of complications, and affordability of care: Except for one woman, all the other respondents gave birth in hospitals or nursing homes. The most common reason for selecting a hospital over home delivery was that facilities were perceived as safer places for emergency care. The perceived quality of care was influenced by a variety of factors especially the need for respectful maternity care. However, family members and neighbors played an important role in forming these perceptions based on their past experiences.

“My first child was born in a government hospital. I had a dreadful experience there. My first child was delivered by normal delivery, and the labor room staff was really hostile. Despite the fact that the government provides free services, I prefer not to give birth at a government hospital since you will not be treated with dignity.” (P14, Village-B, 28 yrs, second gravida) 

Several participants who underwent delivery at both public and private facilities expressed dissatisfaction with the level of interpersonal care they received, particularly from nurses and other health care personnel with experience of being treated disrespectfully “My first child was born in a government institution, and my second child was born in a private hospital. There are significant differences in patient care. If you have a contact in a government hospital, you will be treated with respect.” (P4, husband, age 31 years.). Fear of infection also influenced respondent perception “There was no one wearing personal protection equipment in this private nursing home. I will never, ever go to the hospital. I was terrified that my child would become infected.” (P24, Village-C, age 24 yrs, primigravida mother) 

Disruption of the primary care service continuum: Another barrier to accessing MCH services was the disruption of linkage with primary care services and reduced attention provided by frontline health workers.

“I delivered during the lockdown. After delivery ASHA did not visit my house but she called me to know the condition of myself and my child.” (P19, Village-B, age 25 yrs, second gravida).

“I moved to my parental house to deliver my baby in a government setting. As a result, my baby missed his initial vaccine doses because the ASHA did not pay a visit to my parent's residence.” (P2, Village-A, age 22 years, second gravida) 

Theme-5: Policy Level Barrier - Challenges and Difficulties With the Conditional Cash Transfer Scheme

The Government of India (GoI) introduced the world’s largest conditional Cash Transfer scheme (CCT) in 2005 known as the Janani Suraksha Yojana (JSY) which incentivizes institutional delivery with the objective of lowering rates of maternal and neonatal mortality. Some participants complained of not having received their JSY payments to date. “I submitted all of the documentation along with the bank account information, but no money has been received yet. I contacted ASHA, who informed me that the program was no longer functioning.” (P9, Village-A, age 23 years, primigravida mother).

## Discussion

This study reports the challenging experiences of recent mothers and their immediate family members in the management of pregnancy and childbirth in a resource-limited, rural, and remote region in India during the COVID-19 pandemic. Our study observed that primigravida mothers were insufficiently educated on the course of pregnancy and potential complications, a finding corroborating previous studies [15,16]. Furthermore, although, all participants were aware of the importance of institutional delivery, deviating from pre-pandemic practice, none of the participants delivered in a government health facility resulting in significant out-of-pocket expenses which were catastrophic in some cases. Previous studies in LMICs have also reported subversion of institutional delivery mechanisms during the pandemic period [17,18].

Poor geographic access and transportation costs have long been recognized as major obstacles to accessing institutional, particularly emergency obstetric care, and a major factor in delay contributing to maternal mortality in LMICs [19]. In this study, fear of COVID-19 infection contributed to suboptimal health-seeking behavior by pregnant women, a finding corroborated in previous studies from LMICs [4,15,20,21]. 

The study findings suggest a significant financial burden on low-income households due to the high out-of-pocket expenditure with concomitant loss of wages during the pandemic. Studies from other states of India and also Africa reported the observation of a similar phenomenon [22,23].

The study evidence indicates that during the pandemic, the high cost of MCH services, the fear of contracting infections, and difficult healthcare provider access in rural and remote regions contributed to an increasing reliance on unlicensed medical practitioners. A similar trend was reportedly observed during previous pandemics such as that of EVD in West Africa [24].

According to this study, the increased household workload was a major factor attributed to the inability of pregnant mothers in accessing recommended MCH services, particularly during periods of lockdown, a phenomenon corroborated by United Nations (UN) women's statistics reports [25]. Household support from mothers-in-law and husbands can potentially influence the health-seeking behavior of pregnant women although the cultural dynamics suggested that the couples were the principal decision-makers in our study [26,27].

This study also did not detect any persistent adverse mental health conditions amongst the PLWs. This is possibly due to family support which is known to be protective [28]. On the contrary, numerous studies have shown that going through life-changing experiences like pregnancy and childbirth exposes women to severe psychological impacts like anxiety and stress, which might increase their risk of post-partum depression (PPD) [29]. However, recent evidence also suggests that the risk of PPD may be similar in both the pandemic and post-pandemic stages [30].

This is the only Indian qualitative study that the researchers could identify that explored and documented the issues experienced by women who experienced pregnancy in geographically isolated locations since the onset of the COVID-19 pandemic. Another strength of this study is the MCH-seeking behavior and their barriers during the pandemic were identified through a comprehensive assessment of perspectives from the entire household comprising women, their husbands, and the mothers-in-law to enable recognition of collective decision-making practices. However, as the study was solely qualitative, it was difficult to quantify the burden of the problem, and the absence of generalizability of study findings is the major limitation. Also, significant recall bias cannot be ruled out as the study interviews were conducted in the period coinciding with the third (Omicron) wave of COVID-19 which was mild compared to the first and especially the second (Delta) wave. Finally, perspectives from the local healthcare providers, community health workers, and health system administrators were not assessed in this study. For instance, we did not corroborate the testimony of the participants regarding the non-disbursement of JSY benefits from the health administration and neither could we assess if the potential lack of mitigation of financial hardship in absence of incentive during the pandemic possibly undermined their antenatal care access.

## Conclusions

The utilization of MCH services adequately is a key element in achieving positive maternal and child health outcomes. Low utilization of MCH services during the pandemic in the study area was recognized as an outcome of individual-level barriers (economic barriers, trust in unlicensed practitioners, poor knowledge and awareness of pregnancy danger signs, increased household responsibility), interpersonal-level barriers and facilitators (household decision-making, family support, fear of contracting infection), and community-level barriers (distance and transportation barriers, movement restriction). 

Future pandemic preparedness must ensure enhanced health policy and administrative focus on preventing significant disruption of MCH services by maintaining improved accessibility to alternative health facilities, monitoring regular home visits by frontline health workers, rendering effective distribution of benefits of social protection schemes such as JSY, and universal promotion of respectful maternity care. Education and sensitization of spouses and mothers-in-law for improving MCH seeking behavior are also needed. Furthermore, health systems in resource-limited settings should strengthen the continuum of care for comprehensive MCH including the assessment of delay in infant and child immunization with catch-up programs, care and support for high-risk infants born during the pandemic, and the continued provision of contraception services including terminal methods.
